# PVT and Vapor Pressure Measurements on Ethane*

**DOI:** 10.6028/jres.080A.006

**Published:** 1976-02-01

**Authors:** G. C. Straty, R. Tsumura

**Affiliations:** Institute for Basic Standards, National Bureau of Standards, Boulder, Colorado 80302

**Keywords:** Density, ethane, vapor pressure, *PVT*

## Abstract

New measurements of the vapor pressures and *PVT* properties of ethane are reported. *PVT* determinations have been made from near the triple point to 320 K at pressures to 33 MPa. The density range investigated extends to more than three times the critical density. The new measurements of the vapor pressures of ethane extend from 160 K to near the critical point.

## 1. Introduction

Liquefied fuel gases, such as LNG, are expected to play an increasing role in satisfying future energy requirements. Accurate thermophysical properties data for these liquefied gas mixtures are necessary for the design of liquefaction plants, transport equipment, shipping and receiving terminals, and for custody transfer. The near infinite variations in mixture compositions encountered with these fuel gases rule out completely experimental or strictly computational approaches for determining these properties. Calculation methods, based on accurate, wide range pure component data and selected mixtures data are being developed in a number of laboratories, and appear to offer the only reliable and economical approach for the generation of the necessary thermophysical properties.

This paper reports new measurements of vapor pressures and *PVT* properties of pure ethane. The measurements have been made as part of a comprehensive program to provide the required experimental data and to develop suitable calculation techniques for mixture properties determinations. *PVT* measurements have been made from near the triple point (90.348 K) [1]^1^ to 320 K at pressures up to 33 MPa. The density range extends to more than three times the critical density. The new measurements of the vapor pressures extend from 160 K to near the critical temperature (305 K).

## 2. Experimental Detail

To measure single-phase densities, the gas expansion technique was used. A series of pressure-temperature observations are made on a nearly constant density sample of fluid confined in a cell of accurately calibrated volume. When either the maximum pressure or maximum temperature is reached, the fluid is expanded, to low pressure, into large calibrated volumes maintained at an accurately known temperature above room temperature. The density can then be determined from the cell volume and the compressibility factor (*PV/RT*) of the ethane at the conditions of the expansion volumes.

The ethane used was commercially available research grade with specified minimum purity of 99.98 percent. This purity was verified by chromatographic analysis. Temperatures were measured on the IPTS (1968) with a platinum resistance thermometer calibrated by the National Bureau of Standards. Pressures above about 3 MPa were measured by referencing to oil pressures derived from an oil dead weight gauge accurate to within 0.015 percent. Lower pressures were measured with a precision fused quartz bourdon tube gauge which had been previously calibrated against an air dead weight gauge accurate to within 0.01 percent. The apparatus and procedures were similar to those used previously in this laboratory for measurements on several other cryogenic fluids [2–5] and have been described in detail [6–8]. Slight modification to existing apparatus was necessary because of the higher critical temperature of ethane. Those external parts of the system which contained fluid during a measurement were heated to well above the critical temperature (typically 330 K) in order to reduce the relative density of the fluid residing in these parts, permitting a more accurate adjusted density to be computed.

## 3. Results

With the techniques used here, each experimental *PVT* “run” consists of a number of pressure-temperature observations lying along a near-isochoric path. About 50 such runs were made covering a density range of from about 1.5 to over 21.5 mol/1. Each run consisted of from 5 to 16 *PVT* points, depending on the density. Measurements were always made at fixed temperatures to permit direct analysis in terms of isotherms. A total of over 450 *PVT* data points was determined. These data are tabulated along isotherms in table 1.

Although comparison with data from other sources is, in general, impossible without multiple interpolations, the agreement has been deduced by examining the density deviations of the various data sets [9, 10] from densities calculated from an equation of state for ethane due to Goodwin [11]. The agreement is found to be, in general, within the combined experimental error. Maximum difference occur in the critical region where the equation of state representation is expected to be less satisfactory and where the experimental densities are subject to increasing uncertainty. Estimated uncertainty in the experimental densities in this work is typically ±0.1 percent at the lowest temperatures, increasing to ±0.2 percent at higher temperatures and lower densities, becoming as much as ±1.0 percent in the critical region.

New vapor pressure measurements also have been made at 5 K intervals from 160 to 300 K and are given in table 2. At each temperature, the pressure was measured at least twice with some ethane being removed from the cell between measurements. Identical pressure observations indicated that the two-phase condition existed in the cell.

A vapor pressure equation of the form

$$ln(P/P_{t})=A_{\chi }+B_{\chi }{\;}^{2}+C_{x}{\;}^{3}+D_{\chi }{\;}^{4}+E_{\chi }{\left(1-\chi \right)}^{3/2}$$ (1)

was fit to all available data for ethane [12]. Here, χ = (1 − *T*_t_/*T*)/(l − *T_t_/T*_c_), and *P* and *T* are the pressure and temperature and *t* and *c* refer to the triple and critical points. Coefficients giving the best fit were found to be the following:

*A* = 10.67324
*B* = 8.33782
*C* −3.08489
*D* =−0.65857
*E* =6.04955
*P_t_* = 1.14 × 10^−5^ bar
*T_t_* = 90.348 *K* [Ref 1]
*T_c_* = 305.330 K [Ref 10]

**Figure 1:**
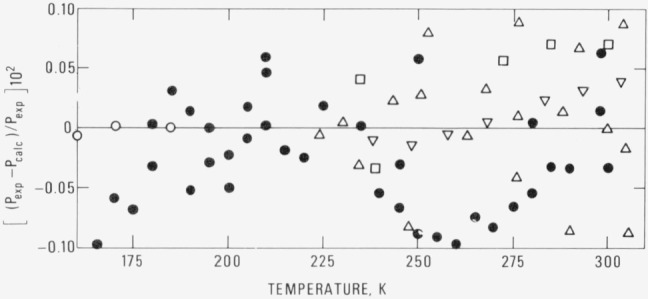
Deviations of vapor pressures from *eq. 1*. ● This work; ○ Ziegler et al. (Ref. [13]); △ Pal (Ref. [9]); □Pope (Ref. [9]); ∇ Douslin and Harrison (Ref. [10]).

Deviations of the experimental vapor pressures from those calculated from this equation for the various data sets [9, 10, 13] are shown in figure 1.

## 4. Summary

We have made new wide-range measurements of the vapor pressures and *PVT* properties of ethane. These are the only data currently available which cover the entire temperature range from the triple point to 320 K. In addition, these data are the only accurate *PVT* data available for the compressed liquid below about 190 K. The data are being used along with other available data to refine the calculation of thermodynamic functions for ethane and as input to, and as a check upon, new calculation methods for predicting liquefied natural (fuel) gas properties being studied in this and other laboratories.

## Figures and Tables

**Table 1:** *PVT* data for ethane

*P* (MPa)		*ρ* (mol/1)
*T*=92.00 K		
0.7928		21.629
*T*=93.00 K		
8.7870		21.682
*T*=94.00 K		
3.5870		21.599
10.4980		21.664
*T*=96.00 K		
6.3091		21.555
14.6217		21.641
*T*=98.00 K		
8.1853		21.503
19.1732		21.625
*T*=100.00 K		
1.7991		21.348
11.3755		21.468
23.8694		21.613
*T*=102.00 K		
4.7964		21.315
15.4890		21.448
28.5382		21.603
*T*=104.00 K		
1.1828		21.198
6.9584		21.269
19.9098		21.434
33.1959		21.594
*T*=106.00 K		
9.0567		21.221
24.4329		21.422
*T*=108.00 K		
1.8227		21.065
6.4514		21.125
12.4806		21.193
29.0045		21.412
*T*=110.00 K		
4.7101		21.034
16.4766		21.176
33.5810		21.403
*T*=112.00 K		
1.1911	20.911	
6.8417	20.990	
11.2851	21.041	
20.6898	21.163	
*T*=114.00 K		
3.8739	20.882	
8.8149	20.942	
24.9715	21.152	
*T*=116.00 K		
1.9636	20.776	
6.3156	20.841	
12.0035	20.913	
19.0660	21.009	
29.2820	21.143	
*T*=118.00 K		
8.0478	20.791	
15.7492	20.896	
23.2017	20.997	
*T*=120.00 K		
1.2747	20.621	
6.8130	20.702	
10.8020	20.757	
19.6919	20.883	
27.3594	20.988	
*T*=122.00 K		
3.8664	20.592	
14.2998	20.737	
23.7193	20.873	
31.5310	20.979	
*T*=124.00 K		
6.2436	20.553	
11.6827	20.627	
18.0492	20.724	
27.7606	20.863	
*T*=126.00 K		
7.9236	20.505	
21.9553	20.712	
31.8410	20.855	
*T*=128.00 K		
0.6715	20.312	
10.4787	20.470	
18.8911	20.597	
25.8617	20.703	
35.9026	20.847	
*T*=130.00 K		
2.9285	20.287	
13.7499	20.450	
29.7809	20.694	
*T*=132.00K		
5.4980	20.254	
17.2765	20.436	
26.4639	20.577	
33.6963	20.687	
*T*=134.00 K		
7.2368	20.209	
20.8906	20.425	
*T*=136.00 K		
1.3689	20.030	
9.2490	20.167	
24.5518	20.416	
34.0682	20.561	
*T*=138.00 K		
3.7831	20.008	
12.1243	20.144	
28.2309	20.407	
*T*=140.00 K		
6.0574	19.972	
15.3375	20.129	
31.9091	20.400	
*T*=142.00 K		
7.6559	19.926	
18.7024	20.117	
35.5822	20.392	
*T*=144.00 K		
0.9387	19.722	
9.8460	19.890	
22.1094	20.107	
*T*=148.00 K		
5.4489	19.667	
15.7717	19.856	
28.9678	20.091	
*T*=152.00 K		
1.2079	19.424	
8.9240	19.584	
22.1746	19.836	
35.8308	20.077	
*T*=156.00 K		
5.5978	19.369	
14.2846	19.545	
28.6589	19.820	
*T*=160.00 K		
0.4675	19.091	
8.9636	19.288	
20.2324	19.524	
35.1468	19.806	
*T*=164.00 K		
4.2580	19.043	
14.0474	19.250	
26.2885	19.508	
*T*=168.00 K		
1.7297	18.817	
7.6437	18.969	
19.6507	19.230	
32.3436	19.495	
*T* = 172.00 K		
1.2449	18.650	
5.7607	18.768	
11.8776	18.916	
25.3400	19.214	
38.3722	19.482	
*T*=176.00 K		
1.1073	18.488	
5.2091	18.602	
8.8759	18.691	
16.9907	18.892	
31.0365	19.201	
*T*=180.00 K		
0.5247	18.295	
4.9583	18.442	
8.2782	18.525	
13.3001	18.655	
22.2633	18.876	
36.7092	19.189	
*T*=184.00 K		
3.9725	18.253	
7.9795	18.368	
12.4806	18.483	
18.2646	18.635	
27.5482	18.862	
*T*=188.00 K		
1.9817	18.021	
7.2058	18.186	
11.9718	18.323	
17.2059	18.463	
23.3273	18.620	
32.8340	18.850	
*T*=192.00 K		
0.9865	17.815	
5.5964	17.977	
10.6669	18.128	
16.5186	18.302	
22.0330	18.447	
28.3464	18.607	
*T*=196.00 K		
0.4943	17.619	
4.4533	17.775	
8.4330	17.903	
14.9662	18.104	
21.1586	18.286	
26.8669	18.434	
33.3677	18.595	
*T*=200.00 K		
0.6801	17.445	
3.5738	17.581	
7.3933	17.709	
12.2367	17.866	
19.4175	18.087	
25.8517	18.273	
31.6945	18.423	
*T*=204.00 K		
3.7760	17.411	
6.7420	17.524	
10.6983	17.658	
16.4076	17.846	
23.9051	18.074	
30.2305	18.262	
36.4896	18.412	
*T*=208.00 K		
0.5897	17.079	
6.8033	17.354	
9.6616	17.461	
14.6253	17.634	
20.6552	17.831	
28.3791	18.062	
35.0871	18.251	
*T*=212.00 K		
3.4269	17.048	
9.6570	17.293	
13.3583	17.434	
18.6673	17.619	
24.9088	17.819	
32.8419	18.051	
*T*=216.00 K		
2.1043	16.798	
6.4050	16.998	
13.2349	17.267	
17.2225	17.417	
22.7370	17.606	
29.1542	17.808	
*T*=220.00 K		
1.4301	16.567	
5.1249	16.761	
8.9985	16.934	
16.9669	17.250	
21.1113	17.404	
26.8053	17.594	
33.3471	17.798	
*T*=224.00 K		
4.3322	16.536	
7.6124	16.699	
12.2714	16.904	
20.7255	17.237	
25.0127	17.392	
30.8565	17.584	
*T*=228.00 K		
1.8552	16.198	
6.9228	16.483	
10.4762	16.654	
15.7164	16.887	
24.4895	17.225	
28.8946	17.382	
34.8859	17.574	
*T*=232.00 K		
1.2667	15.950	
4.5825	16.167	
9.4583	16.428	
13.6904	16.633	
19.2025	16.874	
28.2402	17.215	
32.7523	17.372	
*T*=236.00 K		
3.8508	15.920	
7.0049	16.114	
12.4747	16.403	
16.9765	16.618	
22.6904	16.862	
31.9811	17.205	
*T*=240.00 K		
1.2548	15.515	
2.2021	15.586	
6.3368	15.877	
9.4209	16.062	
15.5972	16.387	
20.2787	16.606	
26.1772	16.852	
35.7046	17.196	
*T*=244.00 K		
3.6366	15.487	
4.6703	15.556	
8.5355	15.817	
12.2246	16.039	
18.7424	16.375	
23.5869	16.595	
29.6476	16.842	
*T*=248.00 K		
1.7380	15.078	
5.9925	15.449	
6.8908	15.508	
11.1353	15.788	
15.1129	16.023	
21.8905	16.364	
26.8798	16.586	
33.1044	16.833	
*T*=252.00 K		
1.5930	14.811	
3.9746	15.056	
8.0325	15.392	
9.0553	15.457	
13.8649	15.771	
18.0296	16.011	
25.0394	16.354	
30.1617	16.577	
*T*=256.00 K		
3.7062	14.790	
6.1397	15.018	
10.3740	15.358	
11.5324	15.433	
16.6249	15.758	
20.9491	16.000	
28.1716	16.345	
33.4255	16.568	
*T*=260.00 K		
2.6858	14.414	
5.7990	14.757	
8.0637	14.963	
12.8526	15.340	
14.0783	15.417	
19.3947	15.747	
23.8662	15.990	
31.2936	16.336	
*T*=264.00 K		
2.4038	14.085	
4.6766	14.389	
7.6561	14.705	
10.2294	14.931	
15.3689	15.327	
16.6466	15.405	
22.1619	15.737	
26.7721	15.981	
34.3986	16.328	
*T*=268.00 K		
3.0548	13.884	
4.2654	14.062	
6.5471	14.352	
9.6680	14.669	
12.5033	14.914	
17.8943	15.316	
19.2306	15.395	
24.9268	15.728	
29.6684	15.973	
*T*=272.00 K		
2.0844	1.243	
3.6257	13.670	
4.8506	13.859	
6.0679	14.032	
8.2942	14.302	
11.8077	14.650	
14.8065	14.901	
20.4232	15.306	
21.8042	15.385	
27.6780	15.719	
32.5542	15.964	
*T*=276.00 K		
2.1376	1.242	
3.2684	13.256	
5.3267	13.644	
6.5457	13.824	
7.7197	13.983	
10.2212	14.275	
13.9836	14.636	
17.1196	14.890	
22.9462	15.297	
24.3837	15.377	
30.4166	15.711	
35.4306	15.957	
*T*=280.00 K		
2.1904	1.241	
2.9758	12.787	
4.8428	13.234	
6.9195	13.604	
8.1526	13.778	
9.4890	13.951	
12.2137	14.259	
16.1653	14.625	
19.4376	14.880	
25.4635	15.288	
26.9473	15.368	
33.1520	15.703	
*T* =284.00 K		
2.2428	1.240	
4.3982	12.768	
6.3596	13.203	
8.4885	13.563	
9.8840	13.751	
11.3395	13.933	
14.2261	14.247	
18.3557	14.615	
21.7505	14.872	
27.9731	15.280	
29.4971	15.360	
35.8724	15.696	
*T*=288.00 K		
2.2951	1.239	
3.0547	1.955	
3.9706	12.194	
5.8026	12.745	
7.7971	13.160	
10.1657	13.540	
11.6682	13.735	
13.2063	13.920	
16.2439	14.237	
20.5477	14.607	
24.0580	14.863	
30.4719	15.273	
32.0446	15.353	
*T*=292.00 K		
2.3466	1.239	
3.8113	11.522	
5.2158	12.173	
7.1396	12.707	
9.3105	13.131	
11.8771	13.525	
13.4688	13.723	
15.0863	13.910	
18.2640	14.228	
22.7394	14.598	
26.3645	14.855	
32.9673	15.265	
*T*=296.00 K		
2.3987	1.238	
3.2373	1.952	
3.8806	2.880	
4.3963	11.212	
4.8686	11.505	
6.4389	12.146	
8.4817	12.672	
10.8697	13.115	
13.6040	13.514	
15.2773	13.713	
16.9735	13.900	
20.2846	14.219	
24.8761	14.591	
28.6475	14.848	
35.4484	15.258	
*T*=300.00 K		
2.4492	1.237	
4.0309	2.875	
4.6932	10.611	
5.3970	11.196	
5.9485	11.485	
7.6275	12.109	
9.8845	12.652	
12.4490	13.102	
12.4489	13.102	
15.3377	13.504	
17.0906	13.704	
18.8621	13.892	
22.3018	14.211	
27.0931	14.583	
30.9501	14.841	
*T*=304.00 K		
2.4997	1.237	
3.4158	1.950	
4.1784	2.873	
4.6828	4.325	
4.7721	9.097	
4.9674	9.788	
5.3930	10.298	
5.5668	10.596	
6.3948	11.173	
6.9986	11.455	
8.8474	12.082	
11.3086	12.638	
14.0362	13.092	
17.0942	13.495	
18.9013	13.696	
20.7454	13.884	
24.3137	14.204	
29.2629	14.576	
33.2309	14.833	
*T*=306.00 K		
4.8094	4.323	
4.9441	6.533	
5.0757	9.092	
5.8014	10.290	
*T*=308.00 K		
2.5499	1.236	
4.3236	2.871	
4.9341	4.321	
5.1434	6.530	
5.3872	9.086	
5.6969	9.775	
6.2111	10.280	
6.4452	10.574	
7.3779	11.142	
8.0352	11.424	
10.0940	12.066	
12.7418	12.628	
15.6258	13.083	
18.6310	13.487	
20.7132	13.688	
22.6296	13.876	
26.3246	14.196	
31.4261	14.569	
35.5024	14.826	
*T*=312.00 K		
2.5996	1.235	
3.5908	1.947	
4.4668	2.869	
5.1793	4.316	
5.5401	6.522	
6.0206	9.072	
6.4330	9.756	
7.0307	10.254	
7.3179	10.546	
8.3744	11.116	
9.1114	11.404	
11.3574	12.054	
14.1819	12.618	
17.2177	13.075	
20.5666	13.480	
22.5240	13.681	
24.5119	13.869	
28.3286	14.189	
33.5824	14.562	
*T*=316.00 K		
2.6491	1.235	
4.6083	2.866	
5.4200	4.312	
5.9358	6.513	
6.6610	9.054	
7.1729	9.732	
7.8532	10.229	
8.1986	10.522	
9.3878	11.099	
10.1982	11.390	
12.6240	12.044	
18.8045	13.068	
22.3004	13.472	
24.3313	13.673	
26.3890	13.862	
30.3236	14.183	
*T*=320.00 K		
2.6983	1.234	
3.7629	1.945	
4.7483	2.864	
5.6574	4.307	
6.3309	6.503	
7.3064	9.032	
7.9172	9.710	
8.6869	10.211	
9.0937	10.506	
10.4137	11.087	
11.2486	11.380	
13.9007	12.036	
17.0663	12.602	
20.4014	13.060	
24.0318	13.466	
26.1304	13.667	
28.2588	13.855	
32.3174	14.176	

**Table 2:** 

*T* (K)	*P* kPa
160.00	21.502
165.00	30.670
170.00	42.870
175.00	58.636
180.00	78.734
180.00	78.706
185.00	103.84
190.00	134.63
190.00	134.72
195.00	172.21
195.00	172.26
200.00	217.26
200.00	217.32
205.00	270.93
205.00	271.00
210.00	334.13
210.00	334.17
210.00	333.98
210.00	333.99
215.00	407.34
220.00	492.16
225.00	589.73
230.00	700.48
235.00	825.96
240.00	966.60
245.00	1124.4
245.00	1124.8
250.00	1300.0
250.00	1301.9
250.00	1302.1
250.00	1301.8
255.00	1495.0
260.00	1670.3
265.00	1947.9
270.00	2208.0
275.00	2493.1
275.00	2493.2
280.00	2804.6
280.00	2806.2
285.00	3144.3
290.00	3513.5
298.15	4190.9
298.15	4188.9
300.00	4353.5

## Footnotes

1 Figures in brackets indicate the literature references at the end of this paper.
